# Cutaneous infection caused by *Mycobacterium szulgai* associated with biologic therapy^؆^

**DOI:** 10.1016/j.abd.2026.501437

**Published:** 2026-08-27

**Authors:** Bárbara Messias Pereira, Bianca Miyazawa, Cássio Rafael Moreira, João Marcos Franco de Souza, Jéssica Pagan Faria, Rogério Nabor Kondo

**Affiliations:** Outpatient Dermatology Clinic, Universidade Estadual de Londrina, Londrina, PR, Brazil

Dear Editor,

Nontuberculous mycobacteria (NTM) are aerobic, acid-fast bacilli found in the environment that, in immunosuppressed individuals, can cause a wide spectrum of infections, including pulmonary and cutaneous forms.^1^ *Mycobacterium szulgai* is an NTM species first described in 1972; it is a rare pathogen responsible for less than 0.5% of all NTM infections.^2^ However, the increasing use of invasive medical procedures and immunobiological therapies has led to a rise in cases caused by these agents.^1^ The present report describes a case of cutaneous *Mycobacterium szulgai* infection in a patient treated with adalimumab for psoriasis and psoriatic arthritis.

The patient was a 45-year-old male mechanic under follow-up at a dermatology clinic for psoriasis and psoriatic arthritis (diagnosed in 2020) affecting his right wrist. He presented with erythematous, scaly plaques on his palms and soles, as well as significant onychodystrophy of the fingernails (Fig. 1), with a Psoriasis Area and Severity Index (PASI) score of 12. The patient had no other comorbidities. Laboratory tests performed prior to the initiation of systemic medications—including complete blood count, renal and hepatic function tests, erythrocyte sendimentation rate (ESR), and C-reactive protein (CRP) were within normal limits. Serology for HIV, syphilis, and hepatitis B and C were non-reactive; interferon-gamma release assays (IGRA) were negative; and chest radiography showed no abnormalities. Consequently, subcutaneous methotrexate (25 mg/mL) was prescribed at a dose of 1 mL/week; however, due to therapeutic failure regarding both cutaneous and articular symptoms, the medication was discontinued after nine months, and the decision was made to introduce an anti-TNF-alpha agent (adalimumab) in January 2021. Two years into this biologic therapy, the patient returned for a consultation presenting with a right-flank ulcer—with serous discharge and a 45-day history—at the medication injection site (Fig. 2A and B).

**Figure 1:**
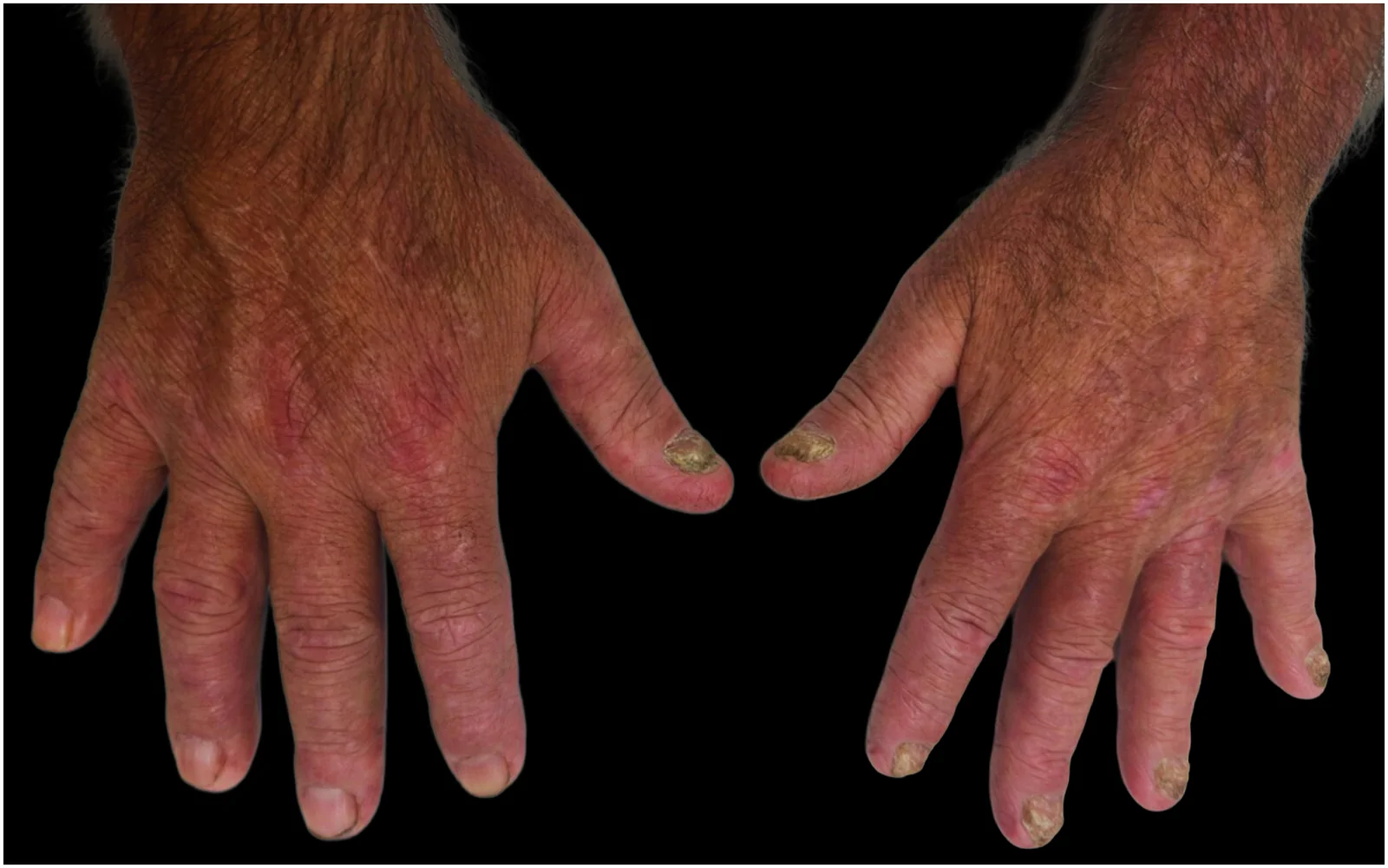
Nail dystrophy of the left hand, characterized by thickening, irregularity, and discoloration of the nails, consistent with nail involvement secondary to psoriasis.

**Figure 2:**
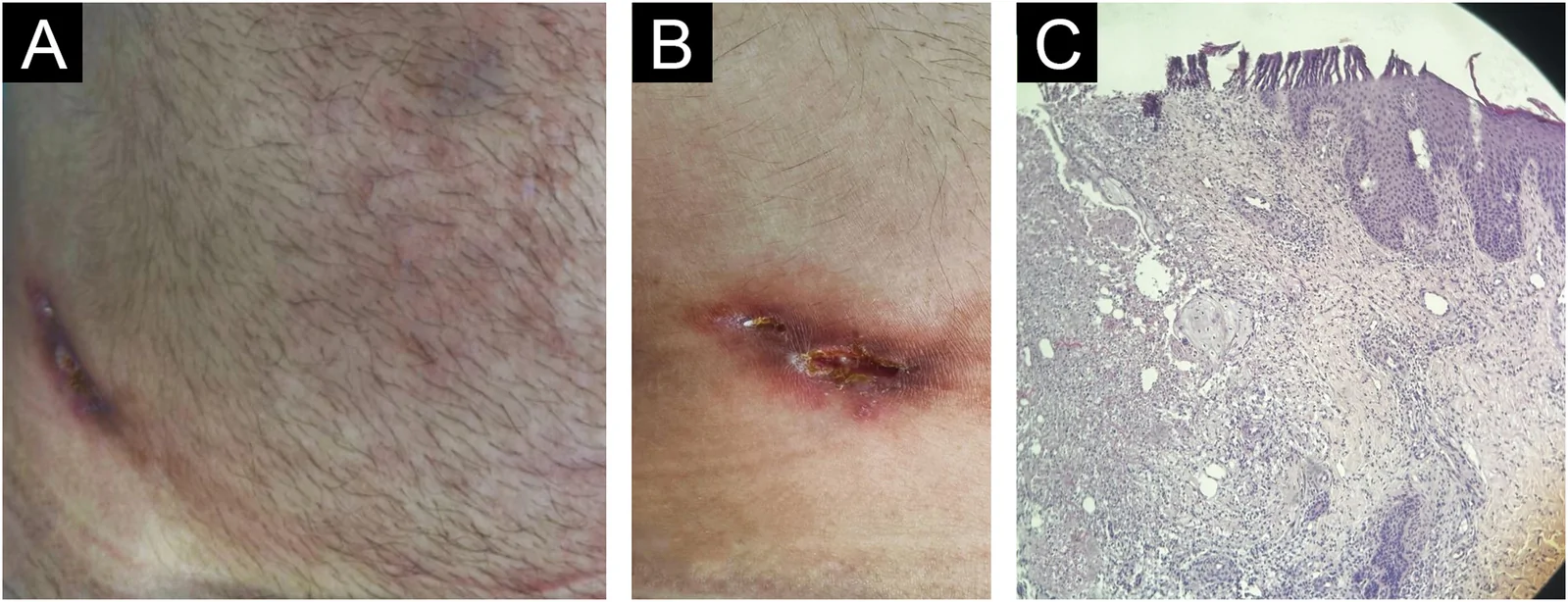
(A) Ulcerated lesion on the right flank at the adalimumab injection site. (B) Detail of the lesion, showing ulceration. (C) Histological section stained with hematoxylin & eosin, ×40 magnification, showing mild reactive epithelial hyperplasia, dermal granulation tissue, and mixed inflammatory infiltrate, with an ulcerated base containing fibrin and neutrophils.

An incisional biopsy was performed, and the skin tissue was sent for histopathological and microbiological analysis (fungi, bacteria, and mycobacteria); findings revealed mild reactive epithelial hyperplasia, dermal granulation tissue, and mixed inflammation involving histiocytes, as well as an ulcer base characterized by fibrin and a neutrophilic infiltrate (Fig. 2C). A skin sample was also tested for acid-fast bacilli (AFB) using the Fite-Faraco stain, yielding a negative result. However, culture analysis revealed the growth of a non-tuberculous mycobacterium identified as *Mycobacterium szulgai* via phenotypic and biochemical methods, as molecular genotyping for species identification was unavailable at the facility.

In this context, adalimumab was discontinued, and treatment for the cutaneous infection was initiated using rifampicin, ethambutol, clarithromycin, and levofloxacin for six months, given that susceptibility testing showed no mutations associated with resistance to these drugs. The patient showed a good response, achieving complete healing of the lesion (Fig. 3). Furthermore, risankizumab was prescribed as a therapeutic option for the underlying dermatological and rheumatological condition, resulting in good control of lesions characteristic of cutaneous, articular, and nail psoriasis, with significant improvement in onychodystrophy (Fig. 4).

**Figure 3:**
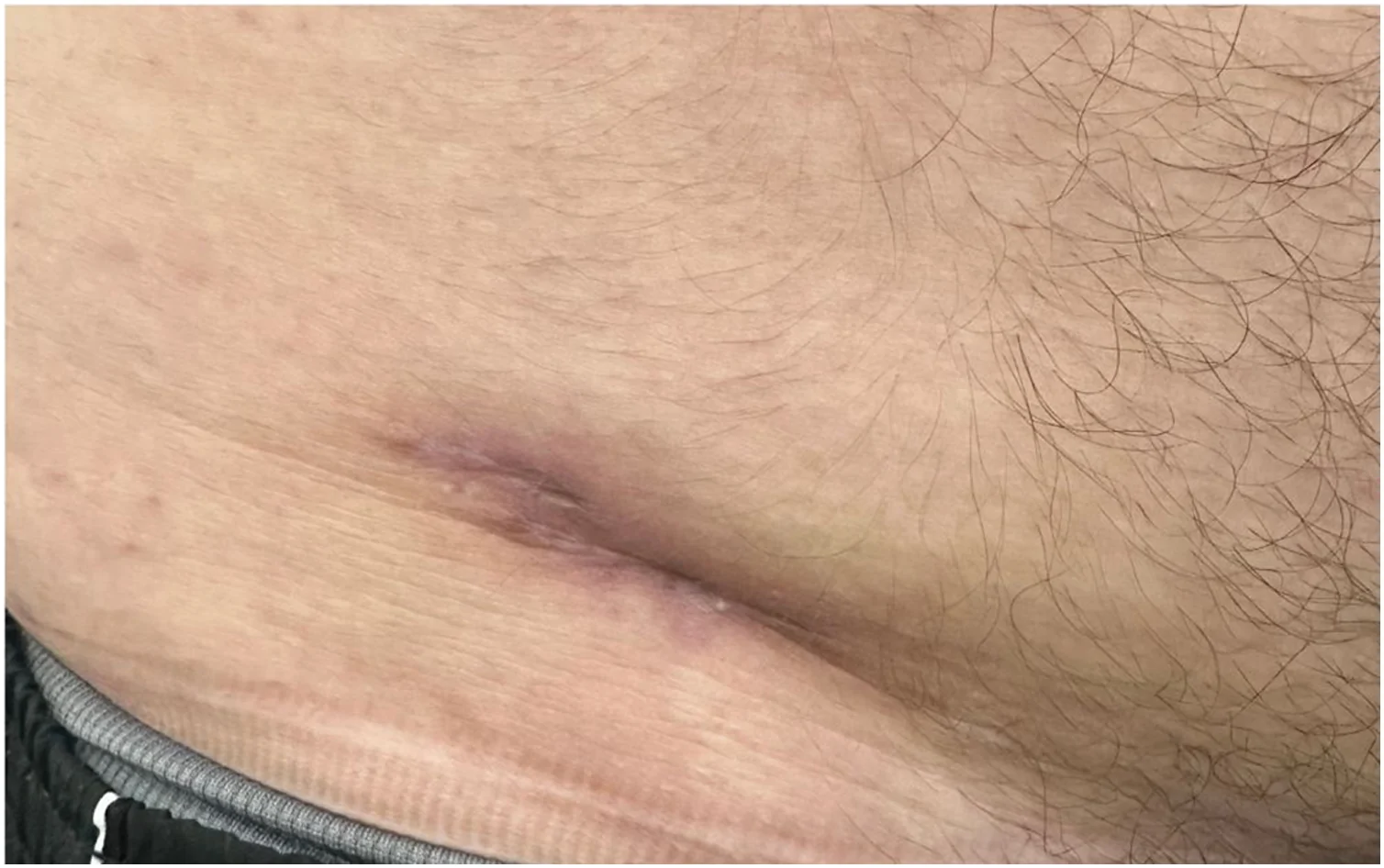
Residual scar following treatment and resolution of the infection.

**Figure 4:**
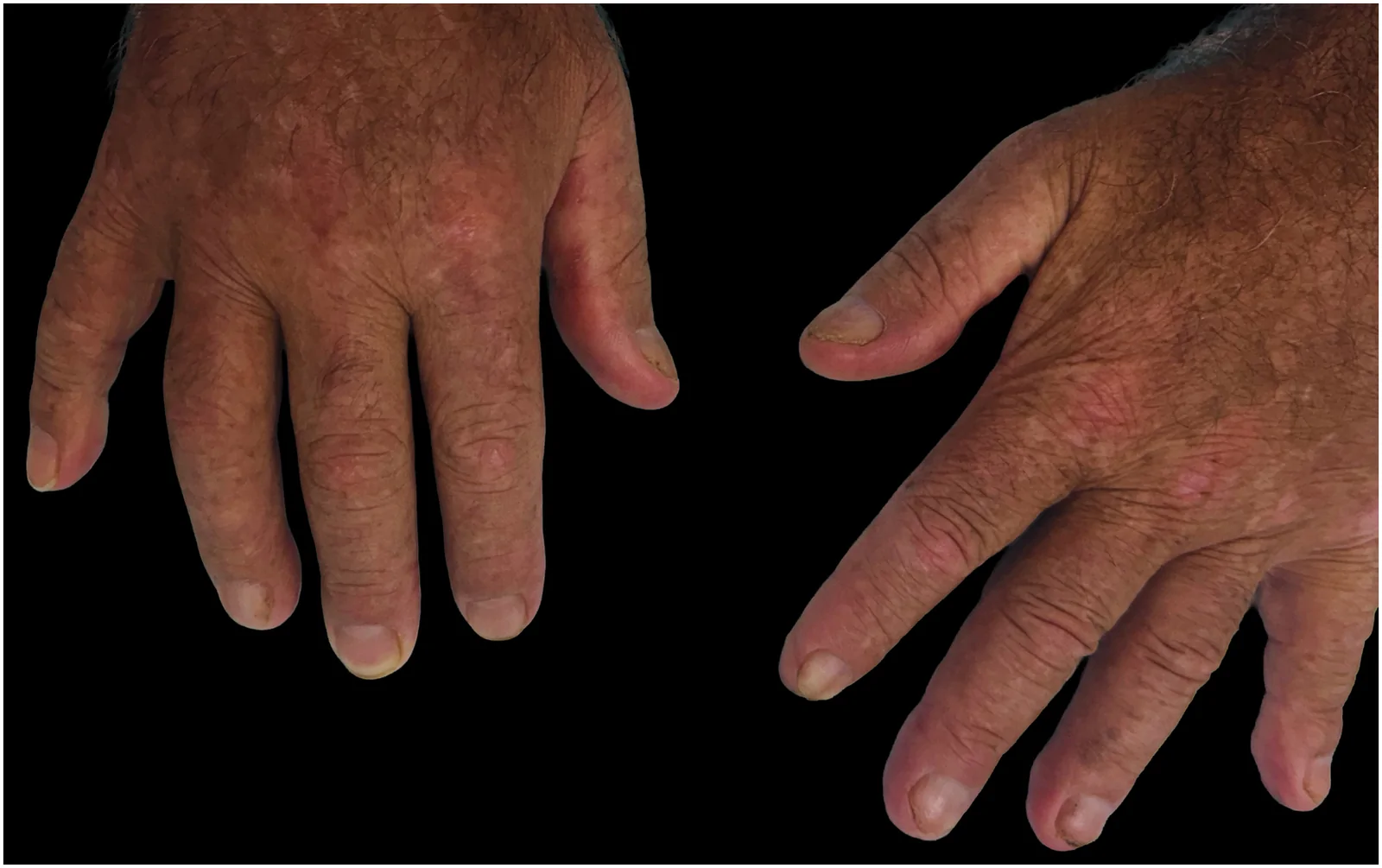
Improvement of onychodystrophy after treatment with risankizumab.

Cutaneous non-tuberculous mycobacterial infections are caused by aerobic acid-fast bacilli that are widely distributed in the environment and responsible for a broad spectrum of infections—most commonly in immunosuppressed individuals. Clinically, they may manifest as pulmonary disease, lymphadenitis, skin or soft tissue infections, bone infections, or disseminated disease.^1^, ^3^, ^4^

The primary species involved in cutaneous disease are *M. marinum*, *M. abscessus*, *M. fortuitum*, and *M. chelonae*; infection typically occurs via direct inoculation following trauma, surgical or aesthetic procedures, or occupational exposure to aquatic environments.^5^ Cutaneous NTM infections present in various forms, such as plaques, papules, pustules, or even subcutaneous nodules, abscesses, and ulcers.^3^

*Mycobacterium szulgai* is a slow-growing NTM species first described in 1972. It is a rare pathogen—accounting for less than 0.5% of NTM infections—that has been isolated from environmental water sources, including hospital tap water, ice machines, fish tanks, and swimming pools. Seventeen cases of cutaneous infection by this agent have been identified in the English-language literature,^2^ while in the Netherlands, *M. szulgai* was isolated from 21 patients between 1999 and 2006.^6^ Two cases of *M. szulgai* infection have been described in Brazil, underscoring the agent's rarity in the country as well as its potential to affect various sites—including skin and soft tissue—with a good response to prolonged antimicrobial treatment.^7^

There is no standard guideline for the pharmacological treatment of this infection; however, most studies have employed regimens containing rifampicin, ethambutol, and clarithromycin—with or without a fluoroquinolone—which have been associated with favorable outcomes.^2^

Anti-TNF-alpha therapy is associated with an increased risk of NTM infections, in addition to tuberculosis (TB). Studies indicate that patients with rheumatoid arthritis (RA) treated with these medications exhibit a higher incidence of these mycobacterial infections.^8^ In a retrospective study involving 239 patients receiving anti-TNF-alpha therapy, Winthrop et al. observed that 105 (44%) met the diagnostic criteria for NTM disease; of these, 73 (69%) were receiving infliximab and 7 (7.7%) were receiving adalimumab. Furthermore, the majority of these patients were concurrently taking other immunosuppressive medications, such as prednisone and methotrexate.^1^

Therefore, it is crucial to monitor patients receiving anti-TNF-alpha therapy for the early detection of NTM infections, particularly in endemic areas and among vulnerable populations.

## ORCID IDs

Bianca Miyazawa: 0009-0003-0561-8504

Cássio Rafael Moreira: 0000-0002-8781-1505

João Marcos Franco de Souza: 0000-0002-9297-7841

Jéssica Pagan Faria: 0000-0001-8727-2348

Rogério Nabor Kondo: 0000-0003-1848-3314

## Research data availability

Does not apply.

## Financial support

None declared.

## Authors' contributions

Bárbara Messias Pereira: Approval of the final version of the manuscript; critical review of the literature; intellectual participation in the propaedeutic and/or therapeutic conduct of the studied cases; critical review of the manuscript; drafting and editing of the manuscript.

Bianca Miyazawa: Approval of the final version of the manuscript; critical review of the literature; intellectual participation in the propaedeutic and/or therapeutic conduct of the studied cases; critical review of the manuscript; drafting and editing of the manuscript.

Cássio Rafael Moreira: Approval of the final version of the manuscript; drafting and editing of the manuscript.

João Marcos Franco de Souza: Approval of the final version of the manuscript; drafting and editing of the manuscript.

Jéssica Pagan Faria: Approval of the final version of the manuscript; drafting and editing of the manuscript.

Rogério Nabor Kondo: Approval of the final version of the manuscript; critical review of the literature; intellectual participation in the propaedeutic and/or therapeutic conduct of the studied cases; critical review of the manuscript; drafting and editing of the manuscript.

## Conflicts of interest

None declared.
